# A proteolytic modification of AIM promotes its renal excretion

**DOI:** 10.1038/srep38762

**Published:** 2016-12-08

**Authors:** Tomoko Yamazaki, Ryoichi Sugisawa, Emiri Hiramoto, Ryosuke Takai, Ayaka Matsumoto, Yoshie Senda, Katsuhiko Nakashima, Peter S. Nelson, Jared M. Lucas, Andrew Morgan, Zhenghua Li, Ken-ichi Yamamura, Satoko Arai, Toru Miyazaki

**Affiliations:** 1 Laboratory of Molecular Biomedicine for Pathogenesis, Center for Disease Biology and Integrative Medicine, Faculty of Medicine, The University of Tokyo, Tokyo 113-0033, Japan; 2 Division of Human Biology and Division of Clinical Research, Fred Hutchinson Cancer Research Center, Seattle, Washington 98109-1024, USA; 3 Center for Animal Resources and Development, Kumamoto University, Kumamoto 860-0811, Japan; 4 AMED-CREST, Japan Agency for Medical Research and Development, Tokyo 113-0033, Japan; 5 Max Planck-The University of Tokyo Center for Integrative Inflammology, Tokyo 113-0033, Japan

## Abstract

Apoptosis inhibitor of macrophage (AIM, encoded by *cd5l*) is a multi-functional circulating protein that has a beneficial role in the regulation of a broad range of diseases, some of which are ameliorated by AIM administration in mice. In blood, AIM is stabilized by association with IgM pentamers and maintains its high circulating levels. The mechanism regulating the excessive accumulation of blood AIM remains unknown, although it is important, since a constitutive increase in AIM levels promotes chronic inflammation. Here we found a physiological AIM-cleavage process that induces destabilization of AIM and its excretion in urine. In blood, IgM-free AIM appeared to be cleaved and reduced in size approximately 10 kDa. Cleaved AIM was unable to bind to IgM and was selectively filtered by the glomerulus, thereby excreted in urine. Amino acid substitution at the cleavage site resulted in no renal excretion of AIM. Interestingly, cleaved AIM retained a comparable potency with full-length AIM in facilitating the clearance of dead cell debris in injured kidney, which is a key response in the recovery of acute kidney injury. Identification of AIM-cleavage and resulting functional modification could be the basis for designing safe and efficient AIM therapy for various diseases.

Apoptosis inhibitor of macrophage (AIM; encoded by *cd5l*)^1^ is a circulating protein belonging to the scavenger receptor cysteine-rich (SRCR) super family, the members of which share different numbers of well conserved cysteine-rich domains (AIM possesses three domains; SRCR1, 2 and 3)^2^,^3^. AIM was initially identified as an apoptosis inhibitor that supports the survival of macrophages against different types of apoptosis-inducing stimuli^1^. AIM is produced by tissue macrophages under transcriptional regulation by nuclear receptor liver X receptor/retinoid X receptor heterodimers^5^,^6^,^7^, and is present at a relatively high level (approximately 5 μg/mL in humans and 2 μg/mL in mice) in blood^4^. In blood, AIM associates with IgM pentamers, which protects AIM from renal excretion and maintains high levels of circulating AIM^8^. Interestingly, in mice and humans with acute kidney injury (AKI), AIM dissociates from IgM pentamers and is excreted in urine. We recently reported that IgM-free urinary AIM accumulates on the AKI-associated intraluminal dead cell debris that obstructs renal proximal tubules and further exacerbates tubular injury^9^. The AIM on the debris interacts with kidney injury molecule 1 (KIM-1; encoded by *havcr1*) expressed on injured tubular epithelial cells^9^, and this response promotes the phagocytic clearance of AIM-bound debris by epithelial cells^9^,^10^,^11^,^12^. Through such a mechanism, AIM contributes to recovery from AKI, and thus, therapeutic AIM administration ameliorated AKI in mice^9^.

We also showed that AIM is incorporated into obese adipocytes and hepatocytes via CD36-mediated endocytosis where it inactivates cytoplasmic fatty acid synthase through direct binding^13^. This response reduces the production of lipid droplet-coating proteins such as fat-specific protein 27 and perilipin, thereby decreasing triacylglycerol deposition within adipocytes and hepatocytes^14^,^15^. This results in the prevention of obesity and liver steatosis, which are hallmarks of metabolic syndrome, which is becoming increasingly prevalent. Interestingly, unlike normal hepatocytes, hepatocellular carcinoma (HCC) cells do not incorporate AIM, but instead, AIM accumulates on their surface. This AIM accumulation inactivates various regulators of complement activation on the surface of HCC cells, thereby provoking complement C3 deposition on the tumour cell surface, leading to necrotic cell death^15^. In accordance, all AIM-deficient (*AIM*^−/−^) mice fed a high-fat diet (HFD) for 1 year developed HCC, whereas no wild-type mice developed the disease^15^. In addition to these diseases, we and others have demonstrated the involvement of AIM in the pathogenesis of a broad range of diseases including *Listeria monocytogenes* infection^5^ and experimental autoimmune encephalomyelitis^16^.

It may be noteworthy that these effects of AIM appear to be achieved in the IgM-independent fashion. During AKI, AIM is released from IgM-pentamers and the resulting IgM-free AIM facilitates the clearance of intraluminal debris at the proximal tubules in kidney^9^. Similarly, AIM alone, not in association with IgM, is incorporated into adipocytes and hepatocytes and promotes lipolysis in obese individuals^13^,^14^,^15^. Also, IgM-free AIM accumulates predominantly on the surface of HCC cells and induces their necrotic death^15^. Thus, it is likely that our body stores a large amount of *inactive* AIM in blood as a complex with IgM pentamers, and AIM is released locally and systemically upon requirement during disease, thereby behaving as active AIM.

While AIM possesses such beneficial roles in defending against different types of disease, we and others also described that a constitutive increase in circulating AIM levels, for example, when fed a HFD, accelerated chronic inflammation^17^ and autoantibody production^8^. In addition, under a cholesterol-rich Western diet, AIM supports the survival of inflammatory macrophages at atherosclerotic regions, resulting in disease acceleration^7^. Such detrimental outcomes of high levels of AIM, which were observed in specific disease models with exaggerated diets, have led us to assess whether certain mechanisms preventing the excess accumulation of blood AIM are present. In this report, we demonstrate a newly discovered proteolytic modification of AIM which may regulate the physiological blood level of AIM, particularly IgM-free active AIM, to avoid undesired disease occurrence.

## Results

### Cleavage of AIM at a specific position during its excretion into urine

**Figure 1:**
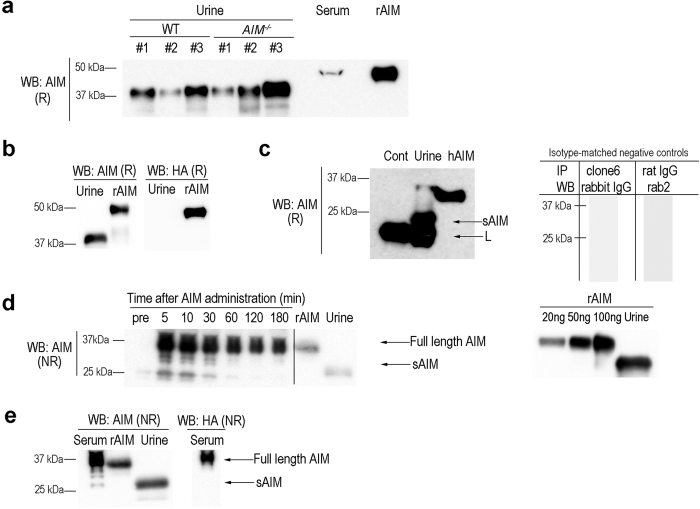
Cleaved AIM appeared in urine. **(a)** Representative immunoblotting (WB) of urine for AIM using an anti-(human & mouse) AIM polyclonal antibody (Rab2). WT (C57BL/6 J) and *AIM*^−/−^ mice were injected with rAIM (200 μg/mouse) intravenously, and urine was collected 1 h after the injection. 1 μL of WT serum and rAIM (20 ng) was loaded as a control. R: reducing condition, NR: non-reducing condition. **(b)** The urine collected from WT mouse 1-2 h after injection with rAIM-HA (200 μg) was analyzed by immunoblotting using the Rab2 antibody or an anti-HA antibody. 3 μl of urine was loaded on each lane. **(c)** Endogenous urinary AIM immunoprecipitated using an anti-human AIM (hAIM) monoclonal antibody (clone #6) from 300 mL of urine collected from a healthy individual, and the precipitant was immunoblotted with Rab2. The human sAIM is indicated by allow. The background light chain of the clone 6 antibody are indicated as L. Immunoprecipitation of sAIM from PBS was also performed (Cont.) As isotype-matched negative controls, immunoprecipitation was also performed using rat IgG followed by immunoblotting using Rab2 antibody, and a set of immuneprecipoitation with clone 6 (anti-human AIM antibody) and immunoblotting with rabbit IgG was also done (right panel). **(d)** Serum from *AIM*^−/−^ mice injected with rAIM (200 μg) intravenously was analyzed for AIM by immunoblotting in a non-reducing condition using Rab2. 1 μL of serum was loaded on each lane. IgM-free full-length AIM (Free AIM) and sAIM are indicated. Immunoblotting of different amounts of rAIM using Rab2 is also presented to confirm that the injected rAIM did not contain sAIM (right panel). **(e)** Immunoblotting of serum from *AIM*^−/−^ mice (5 min after rAIM-HA [200 μg] injection) with Rab 2 or anti-HA. **(d)**, **(e)** Negative control: urine collected from *AIM*^−/−^ mice injected with rAIM.

We previously reported that when mouse recombinant AIM (rAIM) was injected intravenously into mice, rAIM that did not bind to IgM-pentamers was excreted in the urine^8^. Interestingly, we found that the rAIM excreted in urine was reduced in size by approximately 10 kDa compared with the original rAIM as assessed by immunoblotting (Fig. 1a). Identical results were obtained when rAIM was injected into wild-type or AIM-deficient (*AIM*^−/−^) mice (Fig. 1a). When mice were injected with rAIM tagged with an HA-peptide at the C-terminus (rAIM-HA), the cleaved AIM (called small AIM; sAIM hereafter) was detected in urine by an anti-AIM polyclonal antibody that recognizes the first and second cysteine-rich domains, but not by an anti-HA antibody, suggesting that cleavage occurred in the carboxyl-terminus (C-terminus) of AIM (Fig. 1b). We also examined endogenous AIM in urine for cleavage. Since AIM is present at a very low level in healthy humans and mice^9^, we first immuno-precipitated AIM from 300 mL healthy human urine and analyzed it by immunoblotting. The size of the detectable human AIM in urine was also reduced by approximately 10 kDa (Fig. 1c). Although several trials using up to a maximum of 10 mL mouse urine were unable to precipitate urinary AIM at a detectable level by immunoblotting, based on the human data, AIM cleavage appeared to be a physiological consequence along with its excretion in urine.

Next, to address whether AIM was cleaved before or after its excretion in urine, we analyzed sera at different time points after rAIM injection into *AIM*^−/−^ mice. Immunoblotting revealed a smaller AIM in serum that appeared to be the same size with urinary sAIM soon after injection, which decreased and disappeared thereafter (Fig. 1d, Supplementary Fig. 1a,b). Note that the injected rAIM did not contain sAIM (Fig. 1d, right panel). The ratio of sAIM vs. IgM-free AIM (full size), or vs. IgM-bound AIM, reduced rapidly, and was low 60 min after the rAIM injection (Supplementary Fig. 1c). This result is consistent with our observation that a high level of sAIM was detected in urine 60 min after the injection (Fig. 1a). In *AIM*^−/−^ mice injected with rAIM-HA, this smaller AIM in serum was detected by an anti-AIM antibody but not by an anti-HA antibody (Fig. 1e), indicating that this was C-terminus cleaved AIM (i.e. = sAIM). Thus, it is likely that IgM-free AIM was cleaved in blood, and the resulting sAIM was preferentially filtered by the glomerulus and excreted into urine. Intriguingly, despite its cleavage in blood, treatment of rAIM with plasma (prepared with sodium citrate) *in vitro* did not produce sAIM (Supplementary Fig. 2). Since function of some proteases are dependent on Ca^++^, we supplemented calcium chloride in plasma and incubated with rAIM. Again, however, apparent cleavage of rAIM was not observed (Supplementary Fig. 2).

### Putative cleavage site in AIM

**Figure 2:**
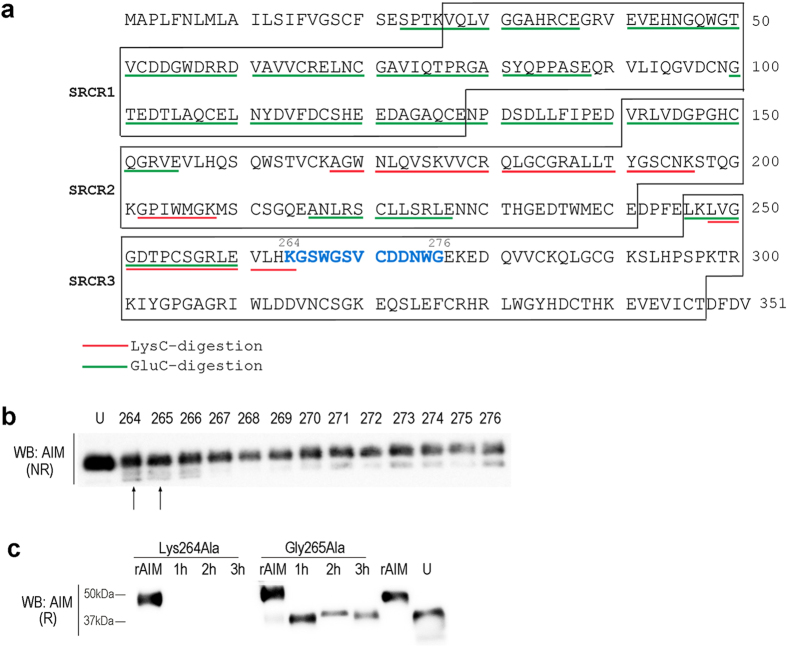
Assessment of the AIM cleavage site. **(a)** LC-MS analysis of urine sAIM fragments after LysC or GluC digestion. Detected fragments were indicated by red lines (LysC digestion) and green lines (GluC digestion) in amino acid sequence of mAIM. The amino acids of the region where the cleavage site may be present are colored by blue. Three SRCR domains are indicated by boxes. **(b)** Culture supernatant of HEK293T cells expressing variant AIM (indicated by amino acid number) and urine collected from *AIM*^−/−^ mice injected with rAIM (200 μg) (indicated by U) were immunoblotted with the Rab2 antibody in a non-reducing condition. The AIM^Lys264^ and the AIM^Gly265^ were present at a comparable position with the urine sAIM (indicated by allows). **(c)** The AIM^Lys264Ala^ and the AIM^265GlyAla^ (200 μg each) were injected intravenously into *AIM*^−/−^ mice and the urine collected after 1, 2, and 3 h was analyzed for AIM by immunoblotting. No sAIM was detected in urine from AIM^Lys264Ala^ injected mice, whereas it was detected in urine from AIM^265GlyAla^ injected mice. rAIM (25 ng) and urine collected from *AIM*^−/−^ mice injected with rAIM (200 μg) (indicated by U) are presented as controls.

We then assessed the cleavage site within AIM. It is well known that amino acid sequence analysis of a protein from the C-terminus is technically difficult, and indeed, we conducted several unsuccessful trials using urinary sAIM purified from rAIM-injected mice. Moreover, intriguingly, the cleaved 10-kDa C-terminus of AIM was not detected in serum or urine: mouse rAIM-HA was injected into mice and their serum and urine were immunoblotted with an anti-HA antibody, but no signal at the corresponding size was detected, suggesting that the cleaved C-terminus might be digested to multiple, undetectable small fragments (data not shown). Thus, it was not possible to use N-terminus sequencing of the C-terminal tail. Instead, therefore, we digested urinary sAIM using the endoproteinases LysC and GluC, and the resulting fragments possessing lysine (Lys) or glutamic acid (Glu) at the C-terminal end were analyzed by liquid chromatography-mass spectrometry (LC-MS). As demonstrated in Fig. 2a, multiple fragments were identified by LC-MS after LysC-digestion (underlined by red). The most C-terminal peptide present was leucine (Leu)^248^-Lys^264^. Similarly, after GluC-digestion, a Leu^246^-Glu^260^ fragment was the most C-terminal peptide (Fig. 2a, underlined by green). From these results, it is most likely that the digested position was located between Lys^264^ and glycine (Gly)^276^ (blue font). We then created variant AIM proteins that terminated at each of the amino acids between Lys^264^ and Gly^276^ (called such as AIM^Lys264^ hereafter) and compared their size with that of urinary sAIM by immunoblotting. We employed a non-reducing condition for SDS-PAGE to preserve possible structural differences that might affect their position on a gel. As demonstrated in Fig. 2b, AIM^Lys264^ and AIM^Gly265^ were detected at approximately similar positions with urinary sAIM, suggesting that AIM might be cleaved at either of these amino acids. Since mouse and human AIM appeared to be cleaved at an identical position based on the observation that the size reduction of AIM after cleavage was similar in mice and humans (Fig. 1a,c), Lys264 and Gly265, which are conserved in human and mouse AIM, were strong candidates for the cleavage site. To this end, we generated variant AIM proteins in which Lys264 or Gly265 was substituted to alanine (AIM^Lys264Ala^ and AIM^Gly265Ala^, respectively), and injected them into *AIM*^−/−^ mice. Immunoblotting of urine from these mice revealed no cleaved (or uncleaved) AIM^Lys264Ala^ protein in urine, suggesting that AIM is cleaved at Lys264 (Fig. 2c). Moreover, the observation that uncleaved AIM^Lys264Ala^ protein did not appear in urine supports the hypothesis that cleaved AIM appeared to be filtered selectively by the glomerulus.

### Uncleaved AIM in urine under ischemia/reperfusion-induced AKI

**Figure 3:**
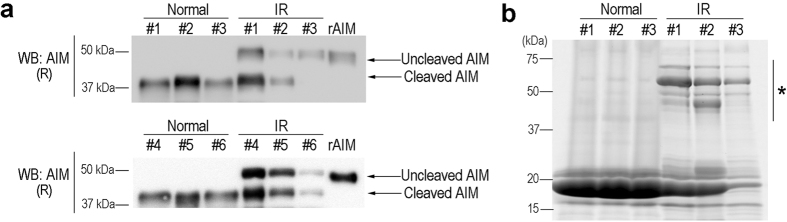
Abrogated preference of sAIM in renal excretion in IR-mice. **(a)** *AIM*^−/−^ mice that had undergone IR (30 min bilateral renal pedicle clamping at 37 °C followed by removal of the clamps) one day before were injected with rAIM (200 μg), and the urine (collected 1 h after the injection) was analysed for AIM by immunoblotting in a reducing condition (n = 6 each). Uncleaved and cleaved AIM were indicated. **(b)** The urine samples from WT mice before and 24 h after IR (three mice) were separated on a SDS-PAGE, and the in-gel proteins were stained by Oriole. *Additional proteins of large molecular weights that appeared in urine from IR-mice.

Recently, we reported that rAIM administered to mice with AKI via intravenous injection was excreted into urine and accumulated on intraluminal debris, and this response facilitated AKI recovery by promoting debris clearance^9^. We wondered whether injected rAIM was also cleaved during its excretion into urine in AKI mice. Therefore, we injected rAIM intravenously into *AIM*^−/−^ mice that had been subjected to ischemia/reperfusion (IR) injury^18^ one day before to induce AKI, and analyzed urine for AIM by immunoblotting. Interestingly, a large proportion of uncleaved AIM was observed in urine from IR-mice (Fig. 3a). The amount of uncleaved AIM was variable in different IR-mice (Fig. 3a), most likely due to the different levels of kidney injury^9^. The urine of IR-mice had more proteins with relatively high molecular weights including albumin, than the urine of healthy mice when assessed using SDS-PAGE (Fig. 3b). Thus, the preferential filtration of cleaved AIM by the glomerulus was abrogated under IR-induced AKI, resulting in excretion of uncleaved AIM in urine.

### AIM cleavage within the urinary tract?

**Figure 4:**
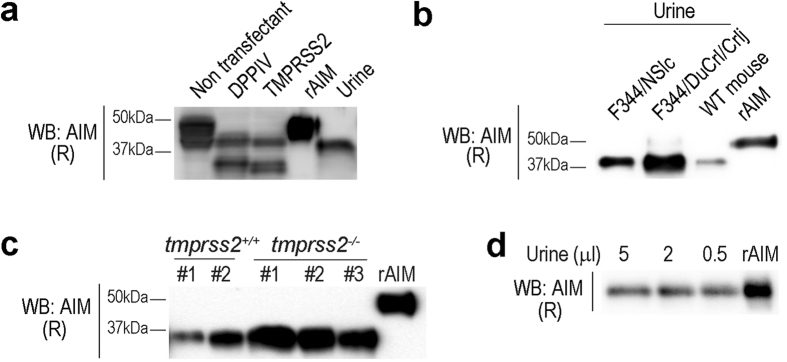
No AIM cleavage in the urinary tract. **(a)** HEK293T cells were transfected with an expression vector of mouse DPPIV or TMPRSS2. Cell lysates were prepared from the transfected and non-transfected cells, and incubated with rAIM (200 ng) for 2 h at 37 °C. The reactants were analysed for cleavage of rAIM by immunoblotting in a reducing condition using the Rab2 antibody. Representative results are presented. Three independent experiments were performed and similar results were obtained. rAIM (100 ng) and the urine collected from *AIM*^−/−^ mice injected with rAIM (200 μg) are presented as controls. **(b)** Mouse rAIM (1 mg) was injected into F344/DuCrl/Crlj and control F344/NSlc rats (n = 2 each). Urine was collected 1 h after the injection, and was analyzed for AIM by immunoblotting using the Rab2 antibody. rAIM (20 ng) is presented as a control. **(c)** rAIM (200 μg) was injected into WT and *tmprss2*^−/−^ mice (n = 3 each), and their urine (collected 2 h after the injection) was analyzed for AIM by immunoblotting. rAIM (20 ng) is presented as a control. **(d)** rAIM (20 ng) was incubated with urine collected *AIM*^−/−^ mice (5, 2, or 0.5 μl) for 2 h at 37 °C and was assessed for cleavage by immunoblotting in a reducing condition. rAIM (20 ng) is presented as a control.

We then assessed if AIM might also be cleaved after glomerular filtration, since it is well known that renal tubules express many proteases^19^,^20^,^21^. Of these, we particularly focused on the serine exopeptidases dipeptidyl-peptidase V (DPPIV, also called CD26)^22^,^23^,^24^ and transmembrane protease, serine 2 (TMPRSS2)^25^, which are apical membrane-bound serine proteases, highly expressing at the brush border of proximal tubular epithelial cells. We first tested whether DPPIV and/or TMPRSS2 cleave AIM. The rAIM protein was incubated with the lysate from HEK293T cells that overexpressed DPPIV or TMPRSS2 for 16 h, and AIM cleavage was assessed by immunoblotting. As presented in Fig. 4a, lysate from DPPIV- or TMPRSS2- expressing HEK293T cells, but not from non-transfected HEK293T cells, cleaved AIM to a size comparable with sAIM. We then studied *in vivo* if AIM-cleavage was abrogated in DPPIV-deficient animals. F344/DuCrl/Crlj is a natural mutant rat deficient for DPPIV^26^. We injected rAIM into F344/DuCrl/Crlj and control F344/NSlc rats that harbors the wild-type *dppiv* allele, and analyzed their urine for AIM by immunoblotting. Note that the AIM amino acid sequence is highly homologous in mice and rats. Unexpectedly, however, only sAIM was detected in urine from F344/DuCrl/Crlj and F344/NSlc rats, indicating that AIM appeared to be cleaved before it encountered DPPIV at the proximal tubules (Fig. 4b). Similarly, rAIM injected into TMPRSS2-deficient mice^27^ was cleaved completely, as in DPPIV-deficient rats (Fig. 4c). Moreover, we treated rAIM with mouse urine, and assessed its cleavage. As shown in Fig. 4d, no rAIM cleavage was induced by incubation with urine. Together, these findings indicate that it is likely that AIM is cleaved in blood before glomerular filtration and not in the urinary tract.

### Abrogated association of sAIM with IgM pentamers

**Figure 5:**
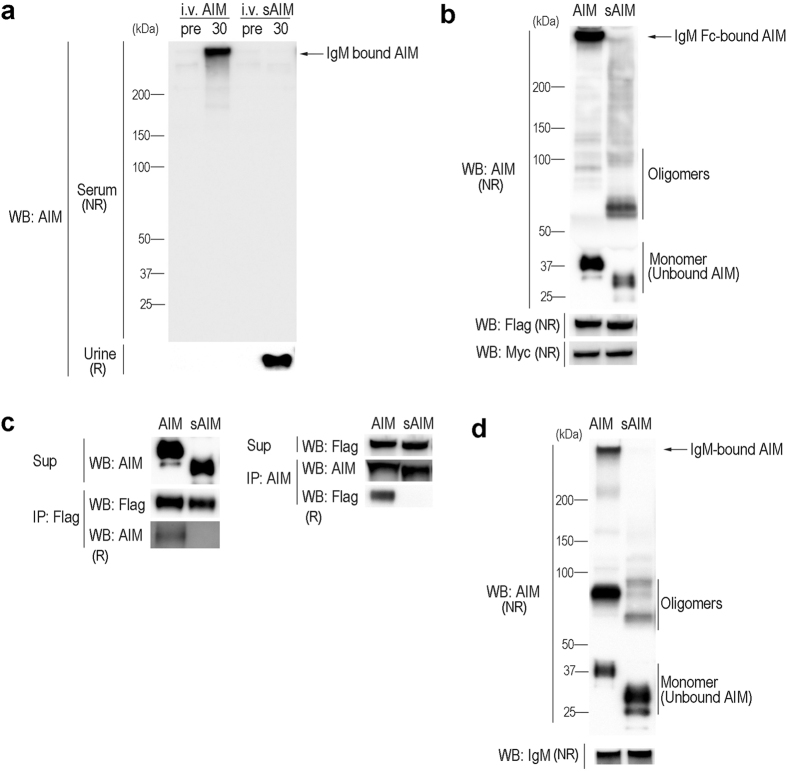
sAIM did not bind to IgM. **(a)** Mouse AIM or sAIM recombinant protein (10 μg) was injected intravenously into *AIM*^−/−^ mice and the serum and urine were tested for AIM by immunoblotting in a non-reducing condition using the Rab2 antibody. “Pre” and “30” indicate the serum and urine collected before and 30 min after AIM injection, respectively. Note that the serum was analyzed in non-reducing (NR) conditions, whereas the urine was analyzed in reducing (R) conditions. Experiments were performed in 3 mice for each protein, and identical results were obtained. **(b)** HEK293T cells expressing AIM or sAIM were co-cultured with the cells expressing FLAG-tagged (at N-terminus) IgM-Fc and Myc-tagged IgJ for 16 h, and the culture supernatant were tested for AIM or sAIM binding to IgM-Fc pentamer by immunoblotting in a non-reducing condition. IgM-Fc-bound AIM, AIM oligomers, and AIM monomer (IgM-unbound AIM) are presented. AIM and sAIM are artificially oligomerized in the supernatants. **(c)** Association of IgM-Fc with AIM but not with sAIM was confirmed by co-immunoprecipitation using the culture supernatants used in **(b)**. The supernatants (indicated by sup) were immunoprecipitated with anti-Flag (left) or anti-AIM (right). **(d)** Recombinant AIM or sAIM was incubated with mouse monoclonal IgM (clone 3F3) protein in PBS for 1.5 h at 37 °C, and their association was assessed by immunoblotting in a non-reducing condition. IgM-bound AIM, AIM oligomers and AIM monomer (IgM-unbound AIM) are presented. AIM and sAIM are artificially oligomerized in PBS.

In healthy mice and humans, AIM associates with IgM pentamers in the blood. Although we showed that AIM binds to the Fc portion of IgM^8^,^28^, the responsible position of AIM for its association with IgM remains unknown. To assess the effect of cleavage on the association of AIM and IgM, we generate recombinant sAIM and tested its association with IgM *in vivo* and *in vitro*. When a small amount (10 μg) of rAIM (which is comparable with the total amount of endogenous serum AIM in wild-type mice) was injected intravenously into *AIM*^−/−^ mice, most of the injected rAIM bound to IgM pentamers and IgM-free AIM was almost undetectable in serum and urine (Fig. 5a). In contrast, the same amount of sAIM injected into *AIM*^−/−^ mice did not bind to IgM pentamers, and the majority was excreted into urine (Fig. 5a). Thus, sAIM lost its ability to associate with IgM. This finding was corroborated *in vitro*. HEK293T cells expressing sAIM or full-length AIM were co-cultured with cells expressing both IgM-Fc and IgJ, and the supernatant was assessed for complex formation by immunoblotting. As demonstrated in Fig. 5b, the association of sAIM with pentameric Fc was entirely abrogated. An immunoprecipitation assay supported this finding (Fig. 5c). Similarly, binding was not seen when sAIM and monoclonal mouse IgM clone (3F3) were incubated in phosphate-buffered saline (Fig. 5d). Thus, it is likely that the responsible site for the association of AIM with IgM is located within the C-terminal tail of the SRCR3 domain. As mouse AIM is *N*-glycosylated at SRCR1 and 2^29^, one might argue that the decrease of molecular weight of AIM during its excretion in urine might be due to deglycosylation. However, it is not likely because the size of injected sAIM was not changed when it appeared in urine (Supplementary Fig. 3). Also, in support, although human AIM is not *N*-glycosylated^29^, the urinary human AIM was also reduced in size (Fig. 1c). As apparent in Supplementary Fig. 1b, when rAIM was injected into *AIM*^−/−^ mice, the amount of IgM-bound AIM did not largely change in 3 h. Since cleaved AIM should dissociate from IgM, this result suggests that IgM-bound AIM appeared to be protected from cleavage.

### Enhancement of debris engulfment by sAIM

**Figure 6:**
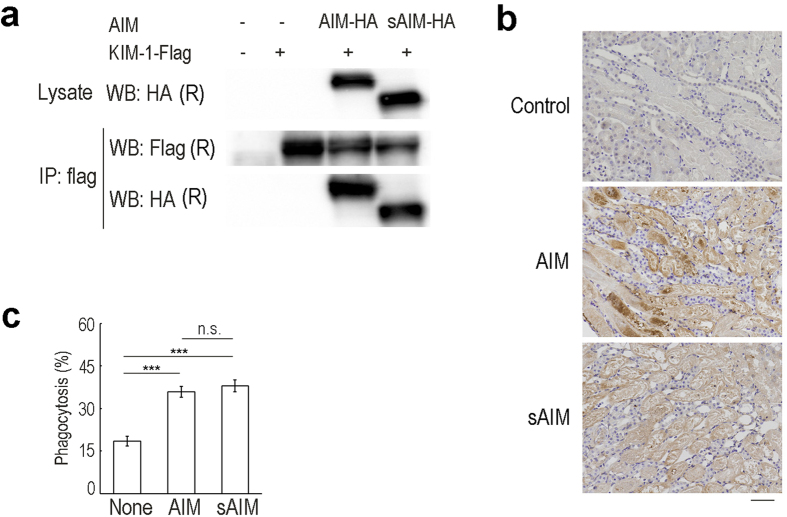
sAIM retained functional association with KIM-1. **(a**) Co-immunoprecipitation assay. AIM or sAIM expression vector was co-transfected with FLAG-tagged KIM-1 expression vector in HEK293T cells, and their association was addressed by immunoprecipitation. Both AIM and sAIM were precipitated with KIM-1-FLAG comparably. **(b)** *AIM*^−/−^ mice that had undergone IR one day before were injected with recombinant AIM or sAIM (200 μg) intravenously. After 1 h, kidney were collected and the specimens were immunostained for AIM. Kidney specimens from IR*-AIM*^−/−^ mice without AIM or sAIM injection were used for control. Scale bar: 50 μm. **(c)** *In vitro* phagocytosis assay. The mProx24 cells with overexpression of KIM-1 were evaluated for engulfment of debris with AIM or sAIM coating, by detection of the eFluor®780 florescence using flow cytometry. The experiment was performed in triplicates, and the percentage (averages ± SEM) of eFluor®780-positive mProx24 cells are shown as Phagocytosis on the graph. ***p < 0.001. n.s.: no significance.

Recently, we reported that AIM binds to KIM-1, which induces the clearance of AIM-bound dead cell debris mediated by KIM-1 expressing proximal tubular epithelial cells, leading to recovery from AKI^9^. We first tested whether sAIM binds to KIM-1. A co-immunoprecipitation assay using HEK293T cells expressing both FLAG-tagged KIM-1 and sAIM revealed that the two proteins co-precipitated each other, indicating that AIM possesses the potential to bind to KIM-1 (Fig. 6a). Judging from the amount of precipitant, the binding efficiency of sAIM and full-length AIM to KIM-1 was comparable (Fig. 6a).

Next, to assess whether sAIM binds to the intraluminal debris that develops in the proximal tubules during AKI in mice, *AIM*^−/−^ mice were subjected to IR, and recombinant sAIM was injected intravenously on day 1 after IR. At 1 h after injection, kidney specimens were immunostained with an anti-AIM antibody. A large proportion of intraluminal debris in the proximal tubules stained positive for sAIM, demonstrating that sAIM also retained the potential to bind to dead cell debris (Fig. 6b).

Having observed that sAIM bound to both KIM-1 and dead cell debris, we then assessed whether sAIM deposition accelerates phagocytosis of dead cell debris by KIM-1-expressing tubular epithelial cells. To this end, we performed an *in vitro* phagocytosis assay. Debris was prepared from necrotic mProx24 cells, a mouse proximal tubular epithelial cell line^30^, and the debris was coated with full-sized AIM or sAIM recombinant protein by co-incubation. Non-coated debris was prepared as a control. The debris was incubated with living mProx24 cells overexpressing KIM-1, and flow cytometry analysis was performed to quantify debris uptake. Debris engulfment by KIM-1-expressing mProx24 cells was comparably increased when the debris was coated with full-sized AIM or sAIM protein compared with non-coated debris (Fig. 6c). Thus, sAIM appeared to be functional in enhancing debris engulfment by KIM-1-positive tubular epithelial cells.

## Discussion

In addition to the therapeutic effect of AIM for AKI which we recently reported, we previously demonstrated that AIM induces lipolysis in adipocytes, thereby regulating obesity^13^. In addition, AIM accumulates on the surface of HCC cells, and induces their elimination via activation of the complement cascade^15^. These findings may strongly inspire a hope that AIM administration could be an effective therapy for different types of disease. Conversely, the constitutively high levels of circulating AIM produced in mice receiving a HFD and/or a high-cholesterol Western diet accelerates chronic inflammation^17^, atherosclerosis^7^, and autoantibody production^8^. These observations may provoke a concern about the therapeutic use of AIM; however, this argument may be dispelled by several findings in our current study. First, we found that IgM-free AIM is cleaved rapidly in blood and preferentially excreted into urine. This mechanism may prevent a constant increase of the levels of blood AIM, in particular of IgM-free functional AIM. Indeed, we analyzed serum AIM levels in more than 8000 humans (including obese ones exhibiting a body-mass-index >25), and none showed such an increase in AIM levels as observed in obese mice fed a HFD and/or Western diet^4^. Second, cleavage of AIM modifies its function. Note that the induction of autoantibody production requires the association of AIM with IgM, which is abrogated in sAIM. Hence, it is unlikely that the therapeutic administration of AIM would cause the excess accumulation of AIM in blood resulting in harmful outcomes. Certainly, further studies are required to clarify whether sAIM might possess a specific, unknown function which might be detrimental to health. It may be noteworthy, however, that general health status including body weight, lethargy, increased eye cloudiness, ruffled fur, and loss of response to tail pain, as well as the levels of various blood markers, were not affected by sAIM injection.

The precise mechanism of how sAIM was selectively filtered by the glomerulus under normal conditions remains unknown. It may simply be selection by molecular size, as uncleaved AIM was also excreted into urine after IR where the size threshold normally present during glomerular filtration appeared to be abolished. Namely, the 10 kDa reduction in its size allows sAIM to pass through the glomerulus. Certainly, one cannot exclude an unknown, active mechanism that avoids the filtration of full-length AIM due to the presence of the 10-kDa C-terminal tail. In addition, the reason why large proteins can pass through the glomerulus after IR needs to be clarified.

The protease responsible for AIM cleavage in blood remains unclear. Various proteases are present in blood, such as coagulation factors and complements. In particular, coagulation factors II, VII, IX, XI, and XII are serine proteases that are constitutively active on the vascular wall, and thus, might be involved in the proteolytic cleavage of AIM in blood. The coagulation pathway is strictly regulated, and each factor has a specific substrate that is usually a different coagulation factor. However, evidence has been accumulating that these factors have extra substrates which are not involved in the coagulation pathway; namely, they may harbor additional substrates not belonging to the coagulation system, thereby contributing to a variety of biological responses. For instance, Shi *et al*. reported that β_2_-glycoprotein I, which is the principle antigenic target of pathogenic antibodies in patients with antiphospholipid syndrome^31^, is an additional substrate of activated factor XI, known to cleave and activate factor IX^32^. Interestingly, activated factor XI cleaves factor IX at arginine (Arg), but cleaves β_2_-glycoprotein I at Lys^31^. Similarly, we found that AIM is cleaved at Lys. However, *in vitro*, rAIM was unable to be cleaved by treating with plasma, even supplemented with Ca^++^ which is required for activation of most coagulation factors. Hence, it might be possible that the AIM-cleavage process cannot be reproduced *in vitro* due to unknown reasons. Thus, in order to identify the responsible protease for AIM-cleavage, further studies are required, particularly *in vivo*, using animals deficient for different types of circulating proteases including coagulation factors.

The sAIM did not bind to IgM, suggesting that the responsible binding site to IgM-Fc should be present in the SRCR3 domain. Although the precise position within SRCR3 that is critical to its association with IgM-Fc still remains unknown, our current findings are useful for its future identification. During AKI, AIM dissociates from IgM and IgM-free AIM levels increase in serum^9^. However, since the increased IgM-free AIM during AKI was full-length, it is unlikely that the AIM cleavage process is involved in its dissociation from IgM. Hence, the precise mechanism responsible for AIM dissociation during AKI remains unknown.

In contrast to its binding with IgM, sAIM retained its ability to bind with KIM-1, suggesting that the region responsible for its binding with KIM-1 may not be present in SRCR3. More importantly, sAIM showed comparable potency to promote debris clearance by epithelial cells through its interaction with KIM-1. Hence, together with the possibility that sAIM may not be trapped by blood IgM, sAIM might be a better form for therapeutic application for AKI than full-length AIM. Further studies to corroborate the superiority of sAIM over full-length AIM as a therapeutic tool, as well as its biological safety, will help to improve the design of AIM-based AKI therapy.

In conclusion, our identification of this AIM-cleavage process could be of help in designing safe and efficient AIM therapy for various diseases.

## Methods

### Animals

*AIM*^−/−^ mice^1^ had been backcrossed to C57BL/6 (B6) for 15 generations before used for experiments. All animal experiments were carried out in strict accordance with the recommendations in the Guide for the Care and Use of Laboratory Animals of the National Institutes of Health. The protocol was approved by the Committee on the Ethics of Animal Experiments of the University of Tokyo (Permit Number: P10–143). The *tmprss2*^−/−^ mice were maintained in Dr. Nelson ‘s laboratory. The F344/DuCrlCrlj rats were purchased from Charles River Laboratories Japan, Inc. and the F344/NSlc rats were purchased from Japan SLC, Inc. Rat experiment was performed in Trans Genic Inc. All surgeries were performed under sodium pentobarbital anesthesia, and all efforts were made to minimize suffering.

### Human subjects

For analysis of human subjects, informed consent in writing was obtained from each healthy volunteer, and the study protocol conformed to the ethical guidelines of the 1975 Declaration of Helsinki as reflected in a priori approval by the Ethics Committee of the University of Tokyo for Medical Experiments (Permission Numbers: #2810, #3358 & #2817).

### Antibodies and reagents

Antibodies and reagents used for histological and biochemical experiments are as follows: Primary antibodies are: AIM (rab2 rabbit polyclonal for mice and human western blot, clones #6 (for human), established in our laboratory, partly purchasable from Trans Genic Inc.), control rabbit IgG (Polyclonal, SIGMA), IgM (Polyclonal, Santa Cruz Biotechnology), HA (clone: 3F10, Roche), FLAG (clone: M2, SIGMA). In addition, Goat anti-Rabbit IgG (H+L) Secondary Antibody, HRP conjugate (Thermo Fisher Scientific), and Hitofine simple stain mouse MAX-PO (R) (NICHIREI, Japan) were used.

### Purification of rAIM (including rAIM-HA and sAIM)

HEK293T cells were transfected with pCAGGS-mouseAIM (with or without HA tag) or pCAGGS-mouse sAIM plasmid and cultured in DMEM, high glucose, GlutaMAX^TM^ medium (Gibco, Carlsbad, CA) supplemented with 10% FBS for 3 days. rAIM was purified from culture supernatant using rat anti-mouse AIM monoclonal antibody (clone #36, house made) conjugated Protein G sepharose (GE Healthcare Life Sciences, PA). Bound protein was eluted with 0.1 M Glycin-HCl pH2.1 and neutralized with 1 M Tris-HCl, pH 8.5. Protein was concentrated as necessary using Amicon Ultra filter concentrators (Millipore, MA), and stored at −80 °C in PBS. Endotoxin levels were measured by the chromogenic LAL endotoxin detection system (Genscript, NJ) following the manufacturer’s protocols. Protein concentration was determined by the BCA (bicinchoninic acid) assay according to the manufacturer’s protocol (Pierce, Rockford, IL).

### *In vitro* AIM cleavage assay

HEK293T cells were transfected with pCAGGS- DPPIV or pCAGGS-TMPRSS2 using Lipofectamine 2000 (Thermo Fisher Scientific). Cells were harvested and lysed in hypotonic buffer (10 mM Tris-HCl, 1 mM MgCl_2_, 0.1% NP-40). After centrifuged 14,000 g for 20 min, supernatant was collected and used for the assay. 100 ng of rAIM was mixed with the lysates containing 200 μg of proteins and incubated for 2 h at 37 °C, and the mixtures were subjected to western blotting to analyse AIM cleavage.

### LC-MS analysis after LysC digestion

200 μg of rAIM was injected into C57BL/6 J mice and their urine was collected 3 h after injection. Urine was concentrated using Amicon Ultra filter concentrators (Millipore, MA) followed by the buffer exchanged into TBS. Proteins in the urine were separated by SDS-PAGE. The gel pieces containing sAIM were alkylated and then digested by LysC or GluC directly. The sAIM fragment proteins were isolated from the digestants, and were analysed by LC-MS using AXIMA®-Performance (Shimadzu Techno-Research, Inc, Kyoto).

### Amino acid substitution in mAIM

Site directed mutagenesis was carried out by PCR using oligonucleotide primers listed below. PCR was performed using the Platinum high-fidelity Taq polymerase (LifeScience Inc.). Resulting PCR products were digested with EcoRI and XhoI, and then subcloned into pCAGGS plasmid. Primers used for this experiment are shown in Supplementary Table 1.

### *In vitro* AIM-IgM association assay (co culture)

HEK 293 T cells transfected with pCAGGS-AIM-HA or pCAGGS-sAIM-HA were co-cultured with HEK 293 T cells transfected with both pCAGGS-FL-mIgM-Fc and pCAGGS-mIgJ-Myc in DMEM, high glucose, GlutaMAX^TM^ medium supplemented with 10% FBS for 2 days. The culture supernatants were analysed by western blotting in non-reducing condition.

### Immunoprecipitation of human urine sAIM

Human urine (300 mL) from a healthy volunteer was concentrated to 1 mL using Amicon Ultra filter concentrators (Millipore, MA). The concentrated urine or PBS as a control was incubated with 5 μg of anti-human AIM antibody (clone #6) conjugated affinity gel (SIGMA) at 4 °C overnight. The precipitates were washed with a wash buffer (1% NP-40 in PBS containing protease inhibitors) for 5 times, and resolved in 20 μl of 1xSDS sample buffer containing methanol. Samples were heated at 95 °C for 5 min, and loaded on a SDS-PAGE for immunoblotting.

### Dead cell debris preparation

The mProx24 cells, a murine renal proximal tubular epithelial cell line derived from C57BL/6 J adult mouse kidney^29^, were kindly provided by Dr. Takeshi Sugaya (CMICS Co., Tokyo), and were maintained in DMEM/F12 (1:1) (Gibco) supplemented with 5 μg/ml insulin, 5 μg/ml transferrin, 5 ng/ml selenous acid, 0.05 μM dexamethasone, 2.5 mg/mL nicotinamide. Cells were heat-killed by incubating at 65 °C for 20 min in PBS and labelled with Fixable Viability Dye (FVD, eBioscience) 780 by incubating for 30 min at 4 °C. Thereafter, dead cells were crushed by pipetting vigorously. For AIM coating, fluorescence-labelled dead-cell debris were incubated in serum-free DMEM/F12 (1:1) containing none (control), AIM or sAIM at concentration of 100 μg/ml at 37 °C for 1 h. AIM coating was confirmed by flow cytometry using anti-AIM antibodies (data not shown).

### Phagocytosis assay (flow cytometry)

The mProx24 cells were transfected with mKIM-1-IRES-EGFP expression vector. 24 h after transfection, cells were mixed with dead cell debris (pre-labelled with FVD eFluor®780) with recombinant AIM or sAIM coating in serum free DMEM/F12 (1:1) supplemented with 5 μg/ml insulin, 5 μg/ml transferrin, 5 ng/ml selenous acid for 30 min at 37 °C. After incubation, cells were harvested, washed with ice-cold PBS 3 times, resuspended in PBS containing DAPI, and were then subjected to flow cytometry (BD LSRII). Live mProx24 cells were identified as DAPI-negative and the cells overexpressing mKIM-1 were determined by EGFP expression. Proportion of engulfment of eFluor®780 (incorporated dead cell signal)-positive dead cell debris in DAPI-negative EGFP-positive mProx24 cells is presented.

### Induction of AKI in mice

IRI induction was performed as previously described^18^. Briefly, mice (male, 8–10 w old) were anesthetized by intraperitoneal avertin injection (250 μg/kg bodyweight), and the both kidneys were exposed through a small flank incision. Both renal arteries and veins were occluded with clamps at 37 °C. After the ischemic period, clamps were released to induce blood reperfusion.

### Histology

AIM or sAIM detection on intraluminal debris was performed as follows. 4 μm sections of PFA-fixed paraffin embedded kidney were immunostained with an rabbit anti-AIM polyclonal antibody (Rab2), followed by incubation with Hitofine simple stain mouse MAX-PO (R) (NICHIREI, Japan) for 30 min. After stained with DAB, sections were counter-stained with hematoxylin. Specimens were analysed using a light microscope: FSX100 (Olympus, Tokyo).

### Statistical analysis

The mean values of data were measured from at least three replicates and ‘Standard Error’ of the means was calculated. Two-way ANOVA was used.

## Additional Information

**How to cite this article**: Yamazaki, T. *et al*. A proteolytic modification of AIM promotes its renal excretion. *Sci. Rep.* **6**, 38762; doi: 10.1038/srep38762 (2016).

**Publisher's note:** Springer Nature remains neutral with regard to jurisdictional claims in published maps and institutional affiliations.

## Supplementary Material

Supplementary Figures

## References

[b1] Miyazaki. T., Hirokami Y., Matsuhashi N., Takatsuka H. & Naito M. Increased susceptibility of thymocytes to apoptosis in mice lacking AIM, a novel murine macrophage-derived soluble factor belonging to the scavenger receptor cysteine-rich domain superfamily. J. Exp. Med. 189, 413–422 (1999). 9892623 10.1084/jem.189.2.413 PMC2192994

[b2] Resnick D., Pearson A. & Krieger M. The SRCR superfamily: a family reminiscent of the Ig superfamily. Trends. Biochem. Sci. 19, 5–8 (1994). 8140623 10.1016/0968-0004(94)90165-1

[b3] Sarrias M. R. . The Scavenger Receptor Cysteine-Rich (SRCR) domain: an ancient and highly conserved protein module of the innate immune system. Crit. Rev. Immunol. 24, 1–37 (2004). 14995912 10.1615/critrevimmunol.v24.i1.10

[b4] Yamazaki T. . Circulating AIM as an indicator of liver damage and hepatocellular carcinoma in humans. PLoS One. 9, e109123 (2014). 25302503 10.1371/journal.pone.0109123 PMC4193837

[b5] Joseph S. B. . LXR-dependent gene expression is important for macrophage survival and the innate immune response. Cell. 119, 299–309 (2004). 15479645 10.1016/j.cell.2004.09.032

[b6] Valledor A. F. . Activation of liver X receptors and retinoid X receptors prevents bacterial-induced macrophage apoptosis. Proc. Natl. Acad. Sci. USA. 101, 17813–17818 (2004). 15601766 10.1073/pnas.0407749101 PMC539759

[b7] Arai S. . A role of the apoptosis inhibitory factor AIM/Spα/Api6 in atherosclerosis development. Cell Metab. 1, 201–213 (2005). 16054063 10.1016/j.cmet.2005.02.002

[b8] Arai S. . Obesity-associated autoantibody production requires AIM to retain IgM immune complex on follicular dendritic cells. Cell Rep. 3, 1187–1198 (2013). 23562157 10.1016/j.celrep.2013.03.006

[b9] Arai S. . Apoptosis inhibitor of macrophage protein enhances intraluminal debris clearance and ameliorates acute kidney injury in mice. Nat. Med. 22, 183–193, (2016). 26726878 10.1038/nm.4012

[b10] Ichimura T. . Kidney injury molecule-1 (KIM-1), a putative epithelial cell adhesion molecule containing a novel immunoglobulin domain, is up-regulated in renal cells after injury. J. Biol. Chem. 273, 4135–4142 (1998). 9461608 10.1074/jbc.273.7.4135

[b11] Ichimura T. . Kidney injury molecule-1 is a phosphatidylserine receptor that confers a phagocytic phenotype on epithelial cells. J. Clin. Invest. 118, 1657–1668 (2008). 18414680 10.1172/JCI34487 PMC2293335

[b12] Yang L. . KIM-1-mediated phagocytosis reduces acute injury to the kidney. J. Clin. Invest. 125, 1620–1636 (2015). 25751064 10.1172/JCI75417 PMC4396492

[b13] Kurokawa J. . AIM is endocytosed into adipocytes and decreases lipid droplets via inhibition of fatty acid synthase activity. Cell Metab. 11, 479–492 (2010). 20519120 10.1016/j.cmet.2010.04.013

[b14] Iwamura Y. . Apoptosis inhibitor of macrophage (AIM) diminishes lipid droplet-coating proteins leading to lipolysis in adipocytes. Biochem. Biophys. Res. Commun. 422, 476–481 (2012). 22579686 10.1016/j.bbrc.2012.05.018

[b15] Maehara N. . Circulating AIM prevents hepatocellular carcinoma through complement activation. Cell Rep. 9, 61–74 (2014). 25284781 10.1016/j.celrep.2014.08.058

[b16] Wang C. . CD5L/AIM Regulates Lipid Biosynthesis and Restrains Th17 Cell Pathogenicity. Cell. 163, 1413–1427 (2015). 26607793 10.1016/j.cell.2015.10.068 PMC4671820

[b17] Kurokawa J. . AIM is required for obesity-associated recruitment of inflammatory macrophages into adipose tissue. Proc. Natl. Acad. Sci. USA. 108, 12072–12077 (2011). 21730133 10.1073/pnas.1101841108 PMC3141977

[b18] Park K. M. . Inducible nitric-oxide synthase is an important contributor to prolonged protective effects of ischemic preconditioning in the mouse kidney. J. Biol. Chem. 278, 27256–2766 (2003). 12682064 10.1074/jbc.M301778200

[b19] Jacquillet G., Rubera I. & Unwin R. J. Potential role of serine proteases in modulating renal sodium transport *in vivo*. Nephron Physiol. 119, 22–29 (2011). 10.1159/000328926 21832858

[b20] Jacquillet G., Rubera I. & Unwin R. J. Potential role of serine proteases in modulating renal sodium transport *in vivo*. Nephron Physiol. 119, 22–29 (2011). 10.1159/000328926 21832858

[b21] Neurath H. Proteolytic enzymes, past and future. Proc. Natl. Acad. Sci. USA. 96, 10962–10963 (1999). 10500108 10.1073/pnas.96.20.10962 PMC34226

[b22] Girardi A. C., Degray B. C., Nagy T., Biemesderfer D. & Aronson P. S. Association of Na(+)-H(+) exchanger isoform NHE3 and dipeptidyl peptidase IV in the renal proximal tubule. J. Biol. Chem. 276, 46671–46677 (2001). 11590171 10.1074/jbc.M106897200

[b23] Augustyns K. . The unique properties of dipeptidyl-peptidase IV (DPP IV/CD26) and the therapeutic potential of DPP IV inhibitors. Curr. Med. Chem. 6, 311–327 (1999). 10101215

[b24] Sedo A., Duke-Cohan J. S., Balaziova E. & Sedova L. R. Dipeptidyl peptidase IV activity and/or structure homologs: contributing factors in the pathogenesis of rheumatoid arthritis? Arthritis Res. Ther. 7, 253–269 (2005). 16277701 10.1186/ar1852 PMC1297595

[b25] Lucas J. M. . The androgen-regulated type II serine protease TMPRSS2 is differentially expressed and mislocalized in prostate adenocarcinoma. J. Pathol. 215, 118–125 (2008). 18338334 10.1002/path.2330

[b26] Matsui T. . Dipeptidyl peptidase-4 deficiency protects against experimental diabetic nephropathy partly by blocking the advanced glycation end products-receptor axis. Lab. Invest. 95, 525–533 (2015). 25730373 10.1038/labinvest.2015.35

[b27] Kim T. S., Heinlein C., Hackman R. C. & Nelson P. S. Phenotypic analysis of mice lacking the Tmprss2-encoded protease. Mol. Cell Biol. 26, 965–975 (2006). 16428450 10.1128/MCB.26.3.965-975.2006 PMC1347042

[b28] Kai T., Yamazaki T., Arai S. & Miyazaki T. Stabilization and augmentation of circulating AIM in mice by synthesized IgM-Fc. PLoS One. 9, e97037 (2014). 24804991 10.1371/journal.pone.0097037 PMC4013091

[b29] Mori M., Kimura H., Iwamura Y., Arai S. & Miyazaki T. Modification of N-glycosylation modulates the secretion and lipolytic function of apoptosis inhibitor of macrophage (AIM). FEBS Lett. 586, 3569–3574 (2012). 23236605 10.1016/j.febslet.2012.08.017

[b30] Kitamura S. . Transforming growth factor-*β* 1 induces vascular endothelial growth factor expression in murine proximal tubular epithelial cells. Nephron Experimental Nephrology. 95, e79–86 (2003). 14610327 10.1159/000073675

[b31] McNeil H. P., Simpson R. J., Chesterman C. N. & Krilis S. A. Anti-phospholipid antibodies are directed against a complex antigen that includes a lipid-binding inhibitor of coagulation: beta 2-glycoprotein I (apolipoprotein H). Proc. Natl. Acad. Sci. USA. 87, 4120–4124 (1990). 2349221 10.1073/pnas.87.11.4120 PMC54059

[b32] Galli M. . Anticardiolipin antibodies (ACA) directed not to cardiolipin but to a plasma protein cofactor. Lancet. 335, 1544–1547 (1990). 1972485 10.1016/0140-6736(90)91374-j

[b33] Shi T. . Domain V of beta2-glycoprotein I binds factor XI/XIa and is cleaved at Lys317-Thr318. J. Biol. Chem. 280, 907–912 (2005). 15522884 10.1074/jbc.M410291200

