# Supplementary material for: A proteolytic modification of AIM promotes its renal excretion
Source: Sci Rep. 2016 Dec 8;6:38762. doi: 10.1038/srep38762 (PMC5144010; doi:10.1038/srep38762)
Supplement: Supplementary Figures [file srep38762-s1.pdf]

Supplementary Figures for

A proteolytic modification of AIM promotes its renal excretion

Tomoko Yamazaki, Ryoichi Sugisawa, Emiri Hiramoto, Ryosuke Takai, Ayaka Matsumoto, Yoshie Senda, Katsuhiko Nakashima, Peter S. Nelson, Jared M. Lucas, Andrew Morgan, Zhenghua Li, Ken-ichi Yamamura, Satoko Arai, Toru Miyazaki

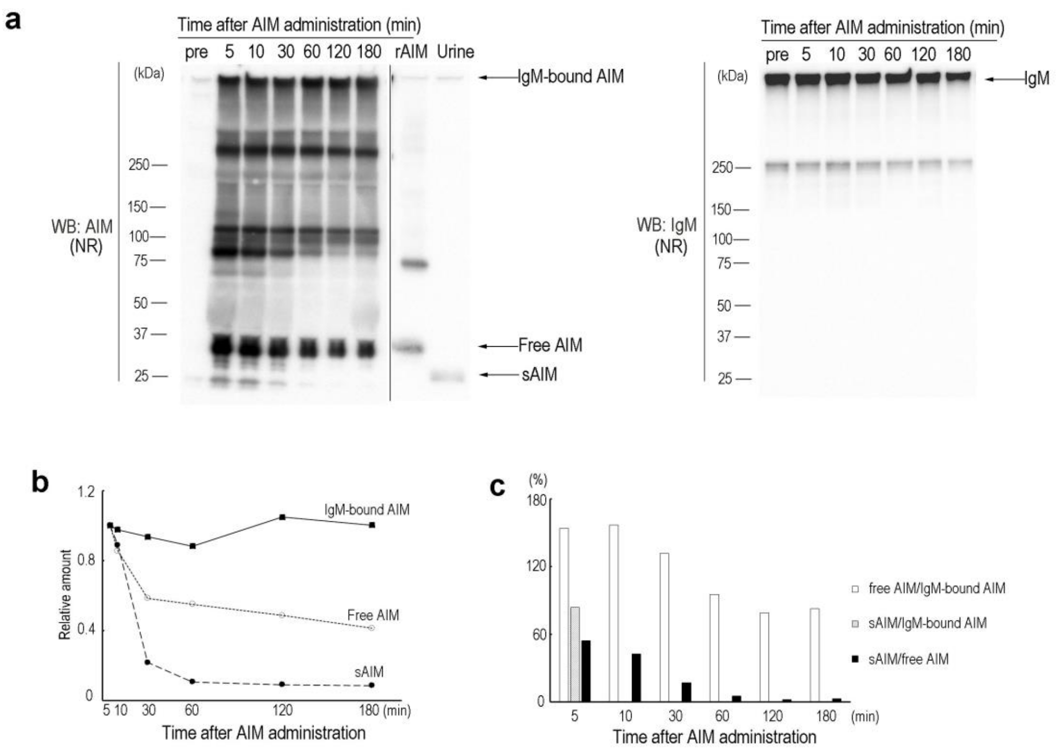

Supplementary Fig. 1. Impaired recover from AKI in *AIM*$^{-/-}$ mice.

(a) The whole image of the immunoblotting presented in Figure 1d. Serum was collected from *AIM*$^{-/-}$ mice injected with rAIM (200 μg) intravenously at different time points and analyzed for AIM by immunoblotting in a non-reducing condition (using Rab2 antibody). IgM-bound AIM, IgM-free full-length AIM (Free AIM) and sAIM are indicated. Some other bands in the blott might correspond to unknown serum proteins that had a potential to associate with AIM. Immunoblotting of the same membrane for IgM in non-reducing conditions (NR) is also presented. **(b)** Amounts of IgM-bound AIM, Free AIM and sAIM at different time points are presented as values relative to those at 5 min after injection. **(c)** The % ration of intensity of free AIM (full length) vs. IgM-bound AIM (white bars), sAIM vs. IgM-bound AIM (shadowed bars), and sAIM vs. free AIM (full length) (black bars). The intensity of the bands for each type of AIM in **(a)** was measured using NIH ImageJ software, and the % ratios at indicated time points are presented by a graph.

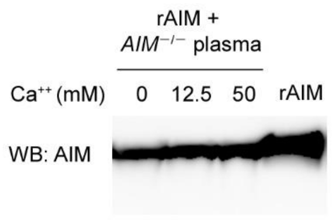

Supplementary Fig. 2. AIM was not cleaved by *in vitro* treatment with plasma. rAIM was incubated with healthy mouse plasma (prepared using sodium citrate) at concentration of final 25 μg/ml with or without calcium chloride (final 12.5 or 50 mM) for 2 h at 37°C, and thereafter each mixture containing 20 ng of rAIM was assessed for the cleavage by immunoblotting with the Rab2 antibody in a reducing condition. rAIM (20 ng) is presented as a control.

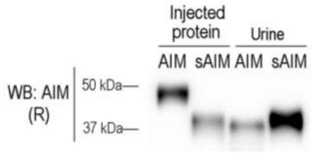

Supplementary Fig. 3. No change of sAIM in size during its excretion in urine. Full-sized mouse AIM or mouse sAIM recombinant protein (200 μg each) was injected into $AIM^{-/-}$ mice, and their urine collected 1 h after the injected was analyzed by immunoblotting using the Rab2 anti-AIM polyclonal antibody in reducing (R) conditions. The injected AIM and sAIM recombinant proteins (50 ng each) were also loaded as controls. Size of AIM was reduced during its excretion in urine, whereas that of sAIM did not change.

Name	Sequence (5'-3')
mAIM Fw	GCGGAATTCCAGCTGCCTGGGCCATGGCTCCAT
Rv 264	GCGAGATCTTCACTTGTGCAGCACCTCCAACCTCCC
Rv 265	GCGAGATCTTCAACCCTTGTGCAGCACCTCCAACCT
Rv 266	GCGAGATCTTCAGGAACCCTTGTGCAGCACCTCCAA
Rv 267	GCGAGATCTTCACCAGGAACCCTTGTGCAGCACCTC
Rv 268	GCGAGATCTTCAGCCCCAGGAACCCTTGTGCAGCAC
Rv 269	GCGAGATCTTCAGGAGCCCCAGGAACCCTTGTGCAG
Rv 270	GCGAGATCTTCAGACGGAGCCCCAGGAACCCTTGTG
Rv 271	GCGAGATCTTCAACAGACGGAGCCCCAGGAACCCTT
Rv 272	GCGAGATCTTCAATCACAGACGGAGCCCCAGGAACC
Rv 273	GCGAGATCTTCAGTCATCACAGACGGAGCCCCAGGA
Rv 274	GCGAGATCTTCAGTTGTCATCACAGACGGAGCCCCA
Rv 275	GCGAGATCTTCACCAGTTGTCATCACAGACGGAGCC
Rv 276	GCGAGATCTTCATCCCCAGTTGTCATCACAGACGGA

Supplementary Table 1. Primers used for amino acid substitution in mAIM.
